# Delayed complete atrioventricular block 11 months after transcatheter aortic valve replacement in a patient with a short membranous septum: a case report

**DOI:** 10.3389/fcvm.2026.1703981

**Published:** 2026-01-30

**Authors:** Jian Xiong, Djandan Tadum Arthur Vithran, Zhixiao Wang, Zheng Cao, Xiaoyong Hu

**Affiliations:** 1 Cardiovascular Disease Diagnosis and Treatment Center, Taihe Hospital, Hubei University of Medicine, Shiyan, Hubei, China; 2 Department of Orthopedics, Xiangya Hospital, Central South University, Changsha, Hunan, China; 3 Department of Cardiology, The Second Affiliated Hospital of Henan University of Science and Technology, Luoyang, Henan, China

**Keywords:** atrioventricular block, case report, membranous septum, permanent pacemaker, transcatheter aortic valve replacement

## Abstract

Conduction disturbances are among the most frequent complications of transcatheter aortic valve replacement (TAVR), typically occurring within the first 72 h after implantation. However, delayed complete atrioventricular (AV) block is rare and may present late with serious clinical consequences. We describe the case of a 72-year-old man with hypertension who developed complete AV block nearly 1 year after TAVR, underscoring the importance of long-term surveillance in patients with high-risk anatomy. The patient initially presented with progressive exertional dyspnea and was diagnosed with severe low-flow, low-gradient aortic stenosis, with a left ventricular ejection fraction of 33%. Preprocedural computed tomography demonstrated a short membranous septum (4.0 mm) and an annulus–membranous septum distance of 1.2 mm, both of which are recognized predictors of conduction disturbances. He underwent transfemoral TAVR with a self-expanding Qiming L26 valve implanted at a depth of approximately 5 mm. Early recovery was uneventful, aside from a small paravalvular leak, which resolved by the 8-month follow-up, at which time echocardiography showed recovery of systolic function to 60%, and sinus rhythm was observed on electrocardiography. At 11 months following TAVR, a community screening ECG revealed 2:1 AV block, and 3 weeks later, he presented with symptomatic complete AV block and a ventricular escape rhythm at 30 bpm. Repeat CT showed increased frame depth (7.6 and 9.8 mm), suggesting possible valve–septum interaction; however, causal attribution is limited by the absence of immediate/serial CT and electrophysiological mapping, and age-related conduction system disease may have contributed to this finding. A dual-chamber permanent pacemaker was implanted, resulting in complete symptomatic recovery and a stable prosthetic valve function. This case report highlights a rare but clinically important phenomenon of very late conduction block after TAVR and supports a risk-stratified approach to anatomical assessment and long-term rhythm monitoring. However, this inference remains hypothesis-generating, given its single-patient nature.

## Introduction

Transcatheter aortic valve replacement (TAVR) has emerged as the standard therapeutic approach for patients with severe symptomatic aortic stenosis, particularly those considered high-risk surgical candidates. Over the past two decades, TAVR has significantly improved outcomes for patients, including symptom relief and improved survival rates (1). However, despite its clinical success, TAVR is associated with a range of complications, notably conduction disturbance. Among these, atrioventricular (AV) block is particularly important, especially in patients receiving self-expanding bioprostheses (1, 2). The mechanism underlying conduction abnormalities is typically attributed to mechanical compression of the His-Purkinje system by the valve frame, which is particularly common in cases involving self-expanding valves. These valves exert a continuous radial force that can disrupt normal electrical conduction in the heart (3, 4). Most conduction disturbances occur in the early periprocedural period, usually within 48–72 h.

Late-onset AV block, particularly beyond 30 days after TAVR, remains a rare and underreported complication (5). Only a limited number of cases describing complete AV block weeks to months following the procedure have been published (1, 3, 6). These disturbances have often been linked to anatomical risk factors, such as a short membranous septum (MS) and deep valve implantation depth, both of which may predispose patients to mechanical compression of the conduction system (4, 7).

We report the case of a 72-year-old man who presented with progressive exertional dyspnea due to severe aortic stenosis and later developed symptomatic complete AV block approximately 11 months after TAVR. This case is unique in documenting the stepwise progression from sinus rhythm to 2:1 AV block and ultimately to complete AV block, while correlating electrocardiographic changes with computed tomography (CT) findings of valve depth and membranous septum length. The resolution of an early paravalvular leak (PVL), which likely reflects continued valve frame expansion, further supports the hypothesis of progressive compression of the conduction system, providing a novel mechanistic link to this delayed complication.

This case underscores the importance of long-term monitoring and careful pre-TAVR anatomical planning, particularly in patients with high-risk features, such as a short MS and deep valve implantation, to mitigate the risk of late-onset conduction disturbance.

## Case presentation

### Patient information

A 72-year-old man with hypertension presented in August 2024 with progressive exertional dyspnea, fatigue, and peripheral edema over several weeks, consistent with a heart failure syndrome. His symptoms developed gradually and worsened over time, significantly limiting his ability to perform his daily activities. He had no history of cardiovascular surgery, syncope, or known conduction disturbances. His medical history was notable for hypertension, which was intermittently controlled with oral antihypertensive therapy. The patient's family history revealed early-onset cardiovascular disease and hypertension in both parents; however, the specific etiology (ischemic vs. structural) could not be verified due to limited medical documentation. The patient had a psychosocial history of moderate stress but no psychiatric conditions or notable genetic concerns. He was a non-smoker and consumed alcohol only occasionally. His physical activity was significantly limited by fatigue and breathlessness. His height was 167 cm and his weight was 56 kg (BMI 20.0 kg/m^2^). On physical examination, the patient appeared to be in no acute distress but exhibited tachycardia with a resting heart rate of 102 bpm and mild bilateral lower extremity edema. His blood pressure was 150/90 mmHg, consistent with his long-standing hypertension. The patient denied chest pain but reported increasing fatigue with mild exertion.

### Clinical findings

On examination, the patient had clinical features consistent with valvular heart failure in the setting of severe low-flow, low-gradient aortic stenosis, including exertional dyspnea, fatigue, tachycardia (heart rate, 102 bpm), elevated blood pressure (150/90 mmHg), and mild bilateral lower-extremity edema. There were no signs of jugular venous distention, pulmonary rales, or peripheral arterial disease. Cardiac auscultation revealed tachycardia heart sounds without a clearly audible systolic ejection murmur, which can occur in severe low-flow, low-gradient aortic stenosis because the reduced forward flow may attenuate the murmurs. Respiratory examination revealed normal breath sounds without signs of respiratory distress. Neurological examination revealed no focal deficits, and the peripheral pulses were intact. Transthoracic echocardiography (TTE) revealed severe low-flow, low-gradient aortic stenosis with an aortic valve area of <1.0 cm^2^, a peak velocity of 2.4 m/s, and a mean gradient of 22 mmHg. The left ventricular ejection fraction (LVEF) was reduced to 33%, consistent with heart failure with reduced ejection fraction. The ascending aorta was dilated to 41 mm, and mild aortic regurgitation was observed. The cardiac chambers were enlarged (left atrium, 45 mm; left ventricle, 61 mm) with mild-to-moderate mitral and tricuspid regurgitation. Bicuspid aortic valve morphology was considered given the ascending aortic dilatation; however, valve morphology was not explicitly documented in the available imaging report text and, therefore, could not be confirmed from the data presented.

The patient was diagnosed with severe aortic stenosis in August 2024, as demonstrated by TTE, and was scheduled for TAVR after assessment by a multidisciplinary team. Table 1 presents the timeline of key events, including the initial presentation, TAVR procedure, and development of AV block at later stages. Following surgery, a small PVL was observed on postoperative day 3 (POD3), which resolved by the 8-month follow-up (May 2025). Follow-up TTE confirmed recovery of left ventricular function, and electrocardiography (ECG) demonstrated sinus rhythm. In July 2025, a community ECG revealed a new 2:1 AV block, and by August 7, 2025, the patient progressed to complete AV block, requiring temporary pacing and ultimately implantation of a dual-chamber permanent pacemaker (PPM).

**Table 1:** Timeline of key clinical events, showing progression from baseline diagnosis through TAVR, delayed AV block, and PPM implantation.

Date	Event description	Diagnostic findings/results	Intervention	Outcome
Aug 2024	Initial presentation with exertional dyspnea. TTE reveals severe low-flow, low-gradient aortic stenosis (AS)	TTE: LVEF 33%, aortic valve area <1.0 cm^2^, peak velocity 2.4 m/s, mean gradient 22 mmHg	N/A	Patient diagnosed with severe aortic stenosis and heart failure
Aug 2024	TAVR procedure with self-expanding Qiming L26 valve. Post-release depth approximately 5 mm. Small paravalvular leak (PVL) noted on POD 3	Post-TAVR TTE: Small PVL observed. No significant changes in valve function at POD 3	TAVR procedure performed with Qiming L26 valve	Small PVL observed, resolved by 8-month follow-up
May 2025	Follow-up TTE shows no PVL and LVEF improved to 60%. ECG shows sinus rhythm at 75 bpm with stable ST-T abnormalities	TTE: No PVL; LVEF 60%. ECG: Sinus rhythm at 75 bpm, stable ST-T abnormalities	Routine follow-up care, including TTE and ECG	LVEF improved, no PVL, stable ECG findings
Jul 2025	Community ECG reveals 2:1 AV block with ventricular rate (VR) 41 bpm	ECG: 2:1 AV block with VR 41 bpm. No significant symptoms reported by patient	No immediate intervention	2:1 AV block detected, but no symptoms reported by the patient
Aug 2025	Complete AV block develops; patient admitted for temporary pacing. Pacing unsuccessful, leading to dual-chamber pacemaker (PPM) implantation	ECG: Complete AV block with VR 30 bpm. TTE: No significant valve dysfunction	Temporary pacing initially, followed by PPM implantation	PPM implantation successful, temporary pacing unsuccessful
Follow-up	PPM functioning well, and patient resumes normal activities with stable TAVR valve function	Device interrogation: PPM functioning well. TTE: Stable TAVR valve function	Ongoing follow-up with device checks and TTE	Patient asymptomatic, normal activity resumed. No adverse events noted

AS, aortic stenosis; AV, atrioventricular; AVA, aortic valve area; CT, computed tomography; EF, ejection fraction; HFrEF, heart failure with reduced ejection fraction; LFLG, low-flow, low-gradient; LVEF, left ventricular ejection fraction; MS, membranous septum; NCC, non-coronary cusp; PPM, permanent pacemaker; PVL, paravalvular leak; TAVR, transcatheter aortic valve replacement; TTE, transthoracic echocardiography; VR, ventricular rate.

### Diagnostic assessment

**Figure 1:**
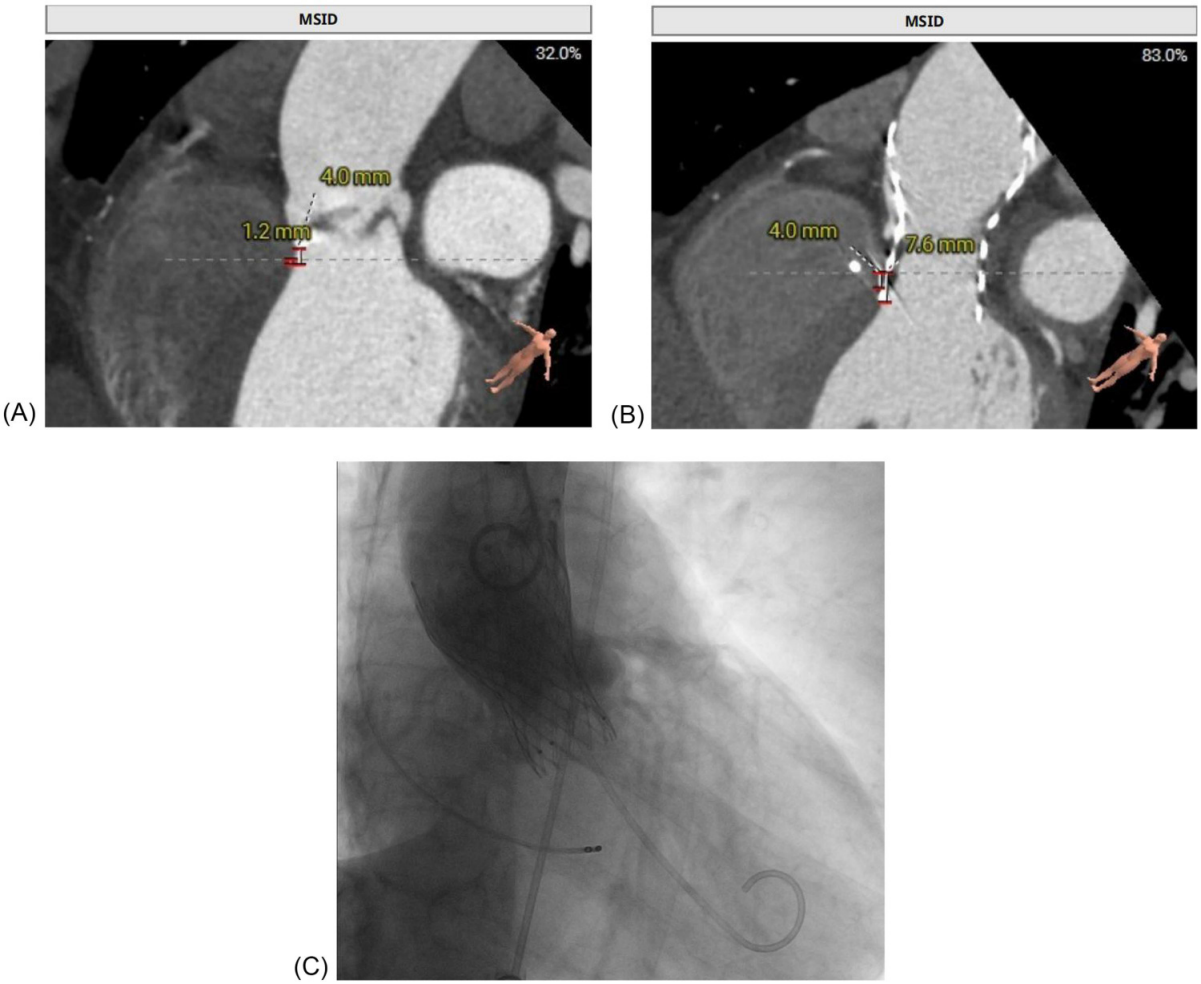
Multimodal imaging of a patient with delayed atrioventricular block after TAVR replacement. (A) Preprocedural CT demonstrated a short membranous septum (4.0 mm) and a membranous septum–annulus distance of 1.2 mm, consistent with a high conduction risk. (B) Post-event CT showed further valve expansion, with a frame depth of 7.6 mm at the membranous septum plane and 9.8 mm at the non-coronary cusp, consistent with progressive compression of the conduction system. (C) Fluoroscopic image during transfemoral TAVR, confirming deployment of the self-expanding Qiming L26 prosthesis at a depth of approximately 5 mm.

**Figure 2:**
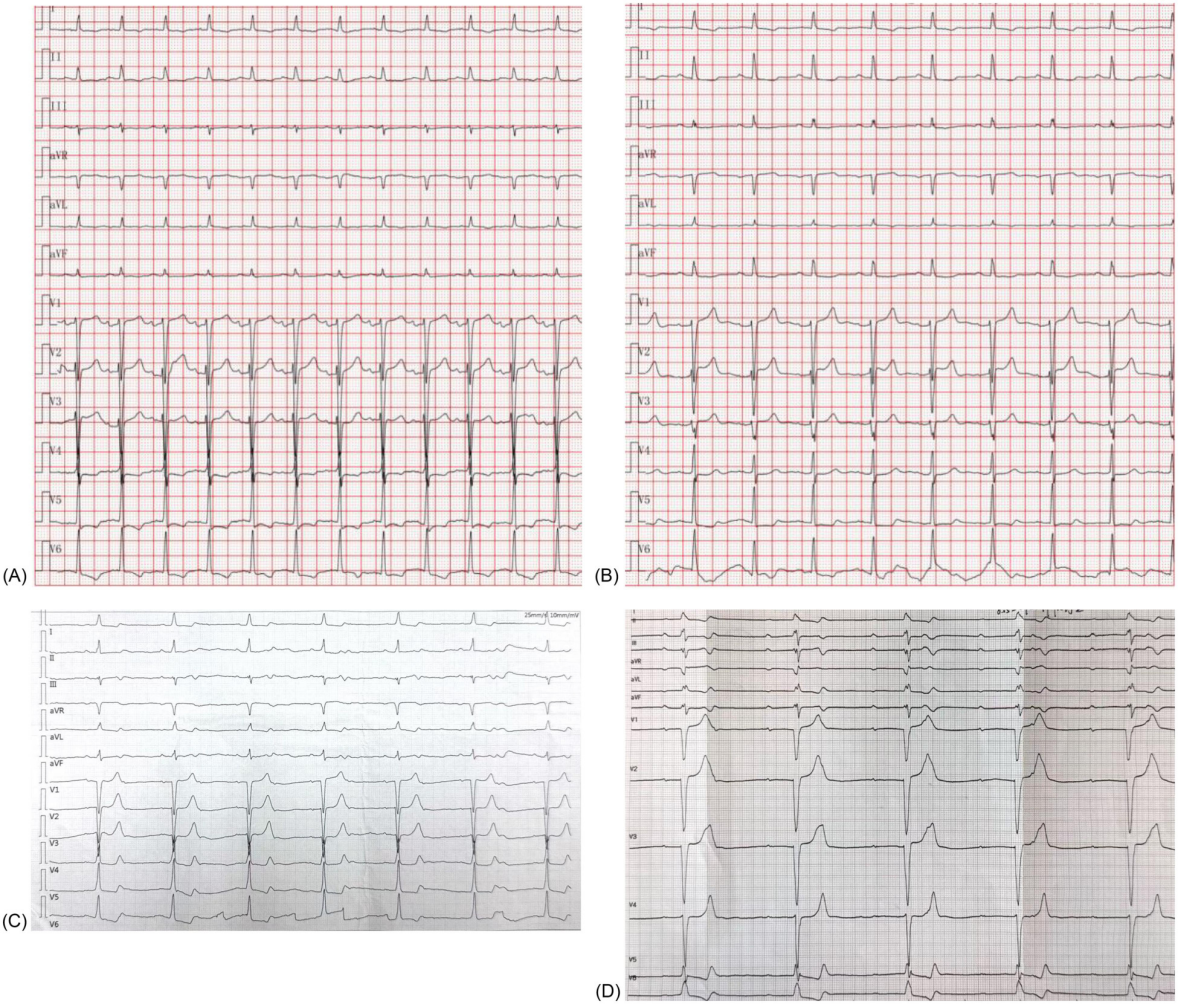
Serial electrocardiograms showing conduction progression after TAVR. (A) Baseline ECG (August 2024) demonstrating sinus tachycardia with non-specific ST–T abnormalities. (B) Follow-up ECG (May 2025) showing sinus rhythm (75 bpm) with persistent ST–T changes but no conduction block. (C) Community ECG (July 2025) demonstrating 2:1 atrioventricular block with a ventricular rate of 41 bpm. (D) Hospital ECG (August 2025) confirming complete atrioventricular block with a ventricular escape rhythm of 30 bpm, necessitating permanent pacemaker implantation.

The patient's presentation with progressive exertional dyspnea and tachycardia was consistent with decompensated heart failure in the setting of severe aortic stenosis. Baseline echocardiography, as detailed earlier, confirmed severe low-flow, low-gradient aortic stenosis with a reduced systolic function. Baseline ECG revealed sinus tachycardia with repolarization abnormalities (ST-segment depression and biphasic/inverted T-waves), fragmented QRS features, and left atrial enlargement (P mitrale) (Figure 2A). Given the patient's age, hypertension, and family history, ischemic cardiomyopathy was considered. Coronary CTA and intraprocedural coronary angiography demonstrated coronary artery disease without vessel occlusion (LM/LAD 60%–70%, LCX 50%, RCA without significant stenosis), and TTE did not show stenosis-correlated regional wall motion abnormality—making an ischemic etiology less likely as the primary driver of reduced LVEF at presentation. Preoperative CT angiography demonstrated a short membranous septum measuring 4.0 mm and a membranous septum–annulus distance of 1.2 mm, findings that placed the patient at an elevated risk for postprocedural conduction abnormalities (Figure 1A). Preprocedural assessment suggested Sievers type I functional bicuspid anatomy with left–right fusion and a heavily calcified raphe. In this context, a balloon-expandable valve was considered but was felt to carry a higher risk of annular/root injury given the calcific burden. Therefore, based on anatomical feasibility and institutional experience, a self-expanding platform was selected. Holter monitoring on postoperative day 2 revealed stable electrical activity without immediate, high-grade conduction disturbances.

### Therapeutic intervention

The patient was admitted for temporary pacing after a diagnosis of complete AV block with a ventricular escape rate of 30 bpm. Despite initial temporary pacing, the block persisted, and after 5 days, a dual-chamber PPM was implanted. A dual-chamber device was chosen over a single-chamber system to preserve atrioventricular synchrony and optimize hemodynamic performance in patients with preexisting left ventricular dysfunction. Conduction system pacing (His-bundle pacing or left bundle branch area pacing) was considered to better preserve ventricular synchrony; however, given the limited local experience with these techniques at the time and the need for prompt, reliable therapy in symptomatic complete AV block, we selected a conventional dual-chamber system with RV septal pacing. Fluoroscopic imaging during TAVR confirmed deployment of the self-expanding Qiming L26 valve at a depth of approximately 5 mm (Figure 1C). Post-implantation TTE showed stable prosthetic valve function, and the patient was discharged in good condition on postoperative day 3.

### Follow-up and outcomes

Our institutional post-TAVR follow-up plan was scheduled at 1, 3, 6, and 12 months with ECG and echocardiography; however, adherence to this plan was incomplete. At the 1-month telephone follow-up, we advised in-person reassessment, but the patient declined the ECG/TTE because he felt well. At the 8-month follow-up in May 2025, TTE demonstrated resolution of the paravalvular leak, and the patient remained in sinus rhythm at 75 beats per minute (Figure 2B). On July 9, 2025, a community ECG revealed a new 2:1 AV block with a ventricular rate of 41 bpm (Figure 2C). As the patient reported no marked symptoms at that time and did not appreciate the risk, he did not seek urgent specialist assessment at that time. Three weeks later, on August 7, 2025, he presented with progressive fatigue and dizziness, and a hospital ECG confirmed complete AV block with a ventricular escape rhythm of 30 bpm (Figure 2D). Repeat CT at this time showed progressive valve expansion, with a frame depth of 7.6 mm at the membranous septum plane and 9.8 mm at the non-coronary cusp, consistent with mechanical compression of the conduction system (Figure 1B). Device interrogation confirmed appropriate dual-chamber pacemaker function, and the patient reported complete symptomatic recovery, resuming normal daily activities with stable prosthetic valve performance.

## Discussion

This case highlights a rare but clinically significant instance of delayed complete AV block occurring 11 months after TAVR in a 72-year-old man with severe aortic stenosis and heart failure with reduced ejection fraction (HFrEF). Although conduction disturbances are recognized as one of the most common complications following TAVR, they typically arise within the first 72 h following the procedure. The unusually late presentation in this patient underscores the importance of extended vigilance, particularly in patients with high-risk anatomical features, such as a short membranous septum and deep valve implantation. In the following sections, we outline the strengths and limitations of this case, synthesize the relevant literature, provide a rationale for our conclusions, and summarize the primary clinical lessons.

### Strengths and limitations in the approach to this case

This case illustrates a rare but clinically significant instance of delayed complete AV block occurring 11 months after TAVR. One of the strengths of this report is the integration of echocardiography, computed tomography with anatomical measurements, serial electrocardiograms, and coronary CTA/angiography, which together support diagnostic reasoning and temporal association. Preoperative CT precisely quantified the MS length at 4.0 mm, and procedural imaging demonstrated a valve implantation depth of 5 mm, both of which are well-established risk factors for post-TAVR conduction disturbance (6–8). This detailed anatomical assessment allowed for a correlation between structural predictors and the eventual development of late conduction abnormalities, thereby providing mechanistic plausibility to the observed outcome. In addition, serial ECGs documented the patient's progression from normal sinus rhythm to 2:1 AV block and ultimately to complete AV block, reinforcing the temporal association between the intervention and the subsequent conduction failure. Timely recognition of conduction deterioration and prompt dual-chamber pacemaker implantation further underscore the clinical value of close monitoring and decisive management.

Another notable strength of this study is the integration of multimodal follow-up. TTE confirmed stable prosthetic valve function and excluded structural complications, whereas clinical evaluation demonstrated symptom relief following pacemaker implantation. Such comprehensive documentation enhances the internal validity of the case and allows for meaningful comparisons with previously published reports.

Nevertheless, important limitations inherent to case reports must be acknowledged. The findings are limited to a single patient and, therefore, cannot be generalized to the broader TAVR population. Moreover, the absence of electrophysiological testing or histopathological confirmation means that the precise mechanism of delayed AV block—whether due to progressive fibrosis, chronic inflammation, or mechanical compression—cannot be elucidated. No electrophysiological study was performed. Although such testing could have assisted in localizing infra-Hisian disease and risk stratification, it was not feasible within the patient's community-to-hospital pathway before the emergent presentation. Similarly, long-term follow-up beyond the initial post-pacemaker period is lacking, precluding conclusions about the impact on survival or heart failure outcomes. Finally, as degenerative conduction disease is common in elderly patients, it cannot be completely excluded as a contributory factor independent of TAVR (6). Cardiac magnetic resonance imaging was not performed prior to TAVR, and no dedicated amyloidosis work-up was undertaken; therefore, an infiltrative cardiomyopathy, such as transthyretin cardiac amyloidosis, cannot be excluded as an alternative or contributory cause of the progressive conduction disease in this patient. In addition, incomplete follow-up adherence likely delayed escalation after community-detected 2:1 AV block, highlighting a real-world gap between recommended surveillance and patient/community pathways. Accordingly, this report should be interpreted as hypothesis-generating. Validation in multicenter registries and comparative cohorts with standardized follow-ups is required before these observations can inform population-level risk prediction or surveillance strategies. These limitations do not diminish the importance of the case but highlight the need for cautious interpretation and the complementary value of larger observational studies.

## Discussion of the relevant medical literature

Conduction disturbances remain among the most common complications following TAVR, and their clinical impacts have been extensively studied. New-onset left bundle branch block (LBBB) occurs in up to 30% of recipients, while high-grade AV block requiring PPM implantation is reported in 7%–24% of cases (1, 5, 9). The majority of these disturbances occur early, typically within 24–72 h after implantation, reflecting acute mechanical compression of the conduction system by the prosthesis frame. Therefore, current guidelines recommend inpatient monitoring with telemetry during the periprocedural period (2).

However, delayed or late-onset AV block, defined as occurring beyond 48 h and sometimes months after the procedure, has gained increasing recognition. Recent studies suggest an incidence of 4%–12%, depending on the population studied and the valve platform used (6, 8). Delayed block is clinically important because it often manifests after discharge, thereby exposing patients to sudden symptomatic bradyarrhythmia or syncope without the safety net of hospital monitoring. In many cases, late AV block has been linked to transient periprocedural conduction disturbances, which later progress, and to self-expanding valves, which exert a chronic radial force on the left ventricular outflow tract and septal tissue (9, 10). Importantly, accumulating evidence indicates that delayed high-grade atrioventricular block after TAVR is not confined to the early periprocedural phase of the procedure. A recent systematic review synthesizing delayed high-grade AV block after TAVR reported a broad incidence range across studies, largely attributable to differences in how “delayed/late” events were defined and how intensively rhythm surveillance was performed (11). Moreover, cohorts using systematic ambulatory rhythm monitoring have demonstrated that clinically important delayed conduction events may be under-ascertained when follow-up is primarily symptom-triggered, supporting a risk-adapted strategy in patients with high-risk anatomy or evolving conduction abnormalities (12).

Anatomical and procedural factors play decisive roles. A short MS places the His bundle in close proximity to the valve frame, increasing susceptibility to injury during implantation. Several studies have shown that an MS length of ≤6 mm is strongly predictive of post-TAVR conduction disturbances and pacemaker need (7, 13, 14). Similarly, deep valve implantation relative to MS increases the likelihood of impingement on the conduction system (15, 16). The present case, characterized by both a short MS and a 5-mm implantation depth, exemplifies this high-risk anatomical constellation in women.

Baseline conduction abnormalities are also important determinants. Preexisting right bundle branch block significantly elevates the risk of complete AV block after TAVR because injury to the left bundle during the procedure leaves no alternative conduction pathway (14, 17). Atrial fibrillation and prolonged PR intervals have also been associated with higher rates of post-TAVR pacemaker implantation (1). In addition, new-onset LBBB and PQ or HV interval prolongation following the procedure predict progression to delayed high-grade block (6, 18).

A summary of published case reports of delayed complete AV block after TAVR is presented in Table 2 (4, 5, 7, 9). While most documented events occurred within 1–3 months, our case is unique in demonstrating progression to complete AV block at 11 months, representing one of the latest presentations reported to date.

**Table 2:** Previously published case reports of delayed complete atrioventricular block after transcatheter aortic valve replacement (TAVR).

Author, year	Journal	Valve type	Timing of AV block	Risk factors noted	Management	Outcome
Rampat et al., 2017	*JACC Cardiovasc Interv*	Self-expanding (CoreValve)	1 month	Deep implantation	PPM	Good
Chen et al., 2022	*J Thorac Cardiovasc Surg*	Self-expanding	Up to 2 months	Short MS length	PPM	Good
Pavlicek et al., 2023	*Clin Res Cardiol*	Self-expanding (Evolut R)	6 weeks	Short MS, diabetes	PPM	Good
Verhemel et al., 2024	*J Cardiovasc CT*	Self-expanding	“Late” conduction events, some >3 months	MS–annulus mismatch	PPM	Mixed

MS,  membranous septum; PPM, permanent pacemaker.

Mechanistically, early conduction disturbances are largely explained by acute mechanical compression, whereas late disturbances may reflect progressive valve expansion, ongoing radial stress from self-expanding devices, or fibrotic remodeling of the conduction tissue over time (3, 9). These hypotheses are supported by imaging and pathologic studies demonstrating valve frame-induced injury to the septal tissue, which may evolve over months. Our case, in which a conduction block developed almost one year following TAVR, aligns with these proposed mechanisms.

Management strategies emphasize early detection and individualized interventions. Intensive ECG monitoring for at least 48–72 h is recommended for all patients, with extended telemetry or ambulatory monitoring advised for those with high-risk features, such as short MS, deep implant, or new-onset conduction abnormalities (2, 9). Several groups have proposed risk-adapted algorithms that incorporate anatomical and procedural parameters, baseline conduction status, and intraprocedural ECG changes to guide monitoring and decision-making (2, 4). Ultimately, persistent or symptomatic high-grade block mandates pacemaker implantation, which remains the definitive treatment. While PPM protects against bradyarrhythmia death, long-term studies suggest that both new-onset LBBB and PPM requirement are associated with higher mortality and heart failure hospitalization, underscoring the prognostic significance of conduction injury (1, 9).

For patients with high-risk anatomy and/or postprocedural electrical changes, a risk-stratified rhythm surveillance strategy is warranted: (i) in-hospital telemetry for ≥48–72 h with repeat 12-lead ECGs; (ii) ambulatory ECG monitoring for 14–30 days when new or progressive conduction abnormalities occur (e.g., new LBBB or significant PR/QRS prolongation); and (iii) scheduled outpatient ECG follow-up at 1, 3, 6, and 12 months and annually thereafter. Pragmatic triggers for urgent reassessment and consideration of pacing include new Mobitz II/2:1 AV block, symptomatic bradycardia, pauses ≥3 s, ventricular rate <40 bpm, or marked PR/QRS prolongation, particularly when accompanied by new LBBB (2, 3).

In retrospect, the community-detected 2:1 AV block (Mobitz II) in this post-TAVR patient should have prompted urgent specialist reassessment and consideration of pacing; earlier escalation might have reduced the likelihood of subsequent symptomatic deterioration. For high-risk patients, strengthened discharge education and remote/wearable heart rate monitoring may improve the early detection of progressive bradyarrhythmias and prompt timely clinical reevaluation.

The conclusions drawn from this case are based on the patient's clinical course and the broader literature. A short membranous septum and deep valve implantation represent well-validated anatomical risk factors for conduction disturbances, as repeatedly confirmed in observational studies and multicenter registries (6, 7, 14, 17). The use of a self-expanding device further increases the risk, as the continuous radial force may account for delayed injury, a mechanism increasingly recognized in recent reports (9, 10). The temporal progression of ECG changes, culminating in complete AV block almost one year later, is biologically plausible and consistent with previously documented cases of late-onset blocks (3, 8).

From a clinical standpoint, this case underscores the need for careful preoperative planning using CT to identify high-risk features and inform decisions regarding valve selection and implantation technique. This highlights the importance of extended post-TAVR surveillance in selected patients. While most conduction disturbances manifest early, late events, although rare, can occur months after the procedure and may be life-threatening if unrecognized. In this context, our case adds to the growing body of evidence advocating risk-based monitoring strategies and individualized follow-up care. The favorable outcome after timely pacemaker implantation further supports guideline-directed management of persistent high-grade block.

Thus, the rationale for our conclusions is twofold: First, anatomical and procedural characteristics can meaningfully predict conduction risk. Second, vigilance beyond the periprocedural period is warranted in high-risk patients. These conclusions align with expert consensus and emerging guidelines (2, 4).

## Conclusion

This case demonstrates that late-onset complete AV block can occur nearly a year after TAVR, particularly in patients with high-risk anatomical features, such as a short membranous septum and deep valve implantation. Comprehensive preoperative imaging, meticulous procedural planning, and individualized postprocedural monitoring are critical for mitigating the risk of delayed conduction disturbances. Vigilance should extend well beyond hospital discharge, and permanent pacemaker implantation remains the definitive therapy for persistent or symptomatic high-grade blocks. The broader lesson is that TAVR, while transformative, continues to carry risks that require ongoing surveillance and patient-specific management strategies. Because this report reflects a single patient, these monitoring suggestions should be interpreted as hypothesis-generating and validated in larger cohorts before being used to inform population-level surveillance protocols.

## Patient perspective

The patient expressed satisfaction with the outcome and relief at being able to resume normal daily activities following pacemaker implantation.

## Data Availability

The original contributions presented in the study are included in the article/Supplementary Material; further inquiries can be directed to the corresponding author.

## References

[1] Auffret V Puri R Urena M Chamandi C Rodriguez-Gabella T Philippon F Conduction disturbances after transcatheter aortic valve replacement. Circulation. (2017) 136(11):1049–69. 10.1161/CIRCULATIONAHA.117.028352 28893961

[2] Lilly SM Deshmukh AJ Epstein AE Ricciardi MJ Shreenivas S Velagapudi P 2020 ACC expert consensus decision pathway on management of conduction disturbances in patients undergoing transcatheter aortic valve replacement. J Am Coll Cardiol. (2020) 76(20):2391–411. 10.1016/j.jacc.2020.08.050 33190683

[3] Rodés-Cabau J Ellenbogen KA Krahn AD Latib A Mack M Mittal S Management of conduction disturbances associated with transcatheter aortic valve replacement. J Am Coll Cardiol. (2019) 74(8):1086–106. 10.1016/j.jacc.2019.07.014 31439219

[4] Verhemel S Nuis RJ van den Dorpel M Adrichem R de Sá Marchi MF Hirsch A Computed tomography to predict pacemaker need after transcatheter aortic valve replacement. J Cardiovasc Comput Tomogr. (2024) 18(6):597–608. 10.1016/j.jcct.2024.08.009 39299898

[5] Rampat R Khawaja MZ Hilling-Smith R Byrne J MacCarthy P Blackman DJ Conduction abnormalities and permanent pacemaker implantation after transcatheter aortic valve replacement using the repositionable LOTUS device. JACC Cardiovasc Interv. (2017) 10(12):1247–53. 10.1016/j.jcin.2017.03.044 28641846

[6] Kikuchi S Minamimoto Y Matsushita K Cho T Terasaka K Hanajima Y Impact of new-onset right bundle-branch block after transcatheter aortic valve replacement on permanent pacemaker implantation. J Am Heart Assoc. (2024) 13(9):e032777. 10.1161/JAHA.123.032777 38639357 PMC11179913

[7] Chen YH Chang HH Liao TW Leu HB Chen IM Chen PL Membranous septum length predicts conduction disturbances following transcatheter aortic valve replacement. J Thorac Cardiovasc Surg. (2022) 164(1):42–51.e2. 10.1016/j.jtcvs.2020.07.072 32891451

[8] Wang G Zhao N Zhang C Zhong S Li X. Lambda-like ST-segment elevation in acute myocardial infarction triggered by coronary spasm may be a new risk predictor for lethal ventricular arrhythmia. Medicine (Baltimore). (2018) 97(49):e13561. 10.1097/MD.0000000000013561 30544473 PMC6310568

[9] Pavlicek V Mahfoud F Bubel K Fries P Ewen S Böhm M Prediction of conduction disturbances in patients undergoing transcatheter aortic valve replacement. Clin Res Cardiol. (2023) 112(5):677–90. 10.1007/s00392-023-02160-0 36680617 PMC10160192

[10] Ueyama H Block PC. Membranous septum length may not be the answer. Catheter Cardiovasc Interv. (2022) 100(5):877–8. 10.1002/ccd.30456 36378723

[11] Rao K Chan B Baer A Hansen P Bhindi R. A systematic review of delayed high-grade atrioventricular block after transcatheter aortic valve implantation. CJC Open. (2024) 6(2):86–95. 10.1016/j.cjco.2023.10.003 38585677 PMC10994975

[12] Ream K Sandhu A Valle J Weber R Kaizer A Wiktor DM Ambulatory rhythm monitoring to detect late high-grade atrioventricular block following transcatheter aortic valve replacement. J Am Coll Cardiol. (2019) 73(20):2538–47. 10.1016/j.jacc.2019.02.068 31118148

[13] Hamdan A Guetta V Klempfner R Konen E Raanani E Glikson M Inverse relationship between membranous septal length and the risk of atrioventricular block in patients undergoing transcatheter aortic valve implantation. JACC Cardiovasc Interv. (2015) 8(9):1218–28. 10.1016/j.jcin.2015.05.010 26292585

[14] Bonnet G Pernot M Zaouter C Peltan J Seguy B Klotz N Membranous septal length and valve implantation depth of TAVR: predictors of new permanent pacemaker implantation after TAVR. Arch Cardiovasc Dis Suppl. (2019) 11(1):70–1. 10.1016/j.acvdsp.2018.10.154

[15] Yushu G Qian X Yanan W Jie L Jingtong W. Prevalence and risk factors of sarcopenia in obese elderly adults. Chinese Gen Pract. (2021) 24(24):3048–53.

[16] Boonyakiatwattana W Maneesai A Chaithiraphan V Jakrapanichakul D Sakiyalak P Chunhamaneewat N Preprocedural and procedural variables that predict new-onset conduction disturbances after transcatheter aortic valve replacement. BMC Cardiovasc Disord. (2022) 22(1):135. 10.1186/s12872-022-02576-y 35361124 PMC8974214

[17] Schamroth Pravda N Shaleve Y Plakht Y Shafir G Grinberg T Wiessman M Interventricular septal thickness on cardiac computed tomography as a novel risk factor for conduction disturbances in patients undergoing transcatheter aortic valve replacement. Europace. (2024) 26(5):euae113. 10.1093/europace/euae113 38691562 PMC11094757

[18] Junquera L Freitas-Ferraz AB Padrón R Silva I Nunes Ferreira-Neto A Guimaraes L Intraprocedural high-degree atrioventricular block or complete heart block in transcatheter aortic valve replacement recipients with no prior intraventricular conduction disturbances. Catheter Cardiovasc Interv. (2020) 95(5):982–90. 10.1002/ccd.28323 31037836

